# The Effects of Spatial Endogenous Pre-cueing across Eccentricities

**DOI:** 10.3389/fpsyg.2017.00888

**Published:** 2017-06-07

**Authors:** Jing Feng, Ian Spence

**Affiliations:** ^1^Department of Psychology, North Carolina State University, RaleighNC, United States; ^2^Department of Psychology, University of Toronto, TorontoON, Canada

**Keywords:** attentional visual field, pre-cueing, endogenous, covert attention, eccentricity

## Abstract

Frequently, we use expectations about likely locations of a target to guide the allocation of our attention. Despite the importance of this attentional process in everyday tasks, examination of pre-cueing effects on attention, particularly endogenous pre-cueing effects, has been relatively little explored outside an eccentricity of 20°. Given the visual field has functional subdivisions that attentional processes can differ significantly among the foveal, perifoveal, and more peripheral areas, how endogenous pre-cues that carry spatial information of targets influence our allocation of attention across a large visual field (especially in the more peripheral areas) remains unclear. We present two experiments examining how the expectation of the location of the target shapes the distribution of attention across eccentricities in the visual field. We measured participants’ ability to pick out a target among distractors in the visual field after the presentation of a highly valid cue indicating the size of the area in which the target was likely to occur, or the likely direction of the target (left or right side of the display). Our first experiment showed that participants had a higher target detection rate with faster responses, particularly at eccentricities of 20° and 30°. There was also a marginal advantage of pre-cueing effects when trials of the same size cue were blocked compared to when trials were mixed. Experiment 2 demonstrated a higher target detection rate when the target occurred at the cued direction. This pre-cueing effect was greater at larger eccentricities and with a longer cue-target interval. Our findings on the endogenous pre-cueing effects across a large visual area were summarized using a simple model to assist in conceptualizing the modifications of the distribution of attention over the visual field. We discuss our finding in light of cognitive penetration of perception, and highlight the importance of examining attentional process across a large area of the visual field.

## Introduction

Expectation about likely locations of a target guides our attention and is essential to efficient identification of important information when interacting with a complex environment (Posner, 1980; Carrasco, 2014; Peelen and Kastner, 2014). For example, when searching for a friend whom one will pick up while driving, the driver allocates attention to the sides of the road rather than the middle of the street. Such expectation, sculpted by target familiarity, memory and scene context (Peelen and Kastner, 2014), is often studied in the laboratory setting using cues indicating the likely location of a target before its presentation. Studies have shown that expectations induced by pre-cues are powerful and operate at very early stages of processing, often even before the stimulus is present (Mangun et al., 1998; Giesbrecht et al., 2006). More specifically, a pre-cue that indicates target locations can enhance spatial resolution at these locations (Yeshurun and Carrasco, 1999), reduce the spatial extent of crowding (Yeshurun and Rashal, 2010), improve perceptual quality (Anderson and Druker, 2013), and affect attentional selection by enhancing the neural response at the locations thus biasing competition favorably toward stimuli at these locations (Kastner and Ungerleider, 2001). This enhancement, as measured by electroencephalogram (EEG) and single cell response, has been seen at both lower and higher levels of the visual cortex including V1 (Motter, 1993), V2 (Motter, 1993; Luck et al., 1997), and V4 (Motter, 1993; Connor et al., 1996; Connor et al., 1997). As a result, information at the expected locations is enhanced, as shown by improved accuracy and faster response speed in identifying a target (Posner, 1980; Carrasco and Yeshurun, 1998; Staugaard et al., 2016).

In the research of pre-cuing effects, several studies reported a significant role of stimulus eccentricity. In one study (Yeshurun and Carrasco, 1998), when attention was drawn by a cue that appeared at the target location before the stimuli display, the pre-cue improved participants’ performance on a texture segregation task in the peripheral locations but impaired task performance at foveal and parafoveal locations (Yeshurun and Carrasco, 1998). Two other studies (Bao and Pöppel, 2007; Bao et al., 2013) examined inhibition of return, an attentional phenomenon that target identification is first enhanced but then impaired by a pre-cue that appeared before the target at the same location, finding significantly stronger inhibition of return in the periphery than in the foveal and perifoveal areas (up to 15° of eccentricity). Functional subdivisions across eccentricities ranging from the foveal to peripheral areas in the visual field have been speculated to be related to the inhomogeneity of the visual field at the physiological and neuroanatomical levels (for a review, see Strasburger et al., 2011), including cortical and subcortical mechanisms (Cowey and Rolls, 1974; Popovic and Sjöstrand, 2001; Bao and Pöppel, 2007; Bao et al., 2013).

Most studies that examined pre-cueing effects on spatial attention have presented stimuli within an eccentricity of around 20°. Very rarely, stimuli were presented outside this area [e.g., Bao et al. (2013) compared attentional processing at 7°–21°]. However, as visual processing starts to show an abrupt change at around 20° of eccentricity, we may not be able to use our understanding of visual attentional processing inside 20° of eccentricity to infer about the processing in more peripheral areas. For example, the velocity of a saccade with an amplitude of up to 20° increases linearly with the amplitude. However, when a saccade’s amplitude goes beyond that, its velocity starts to plateau and the change becomes non-linear with the amplitude (Bahill et al., 1975). In addition, our ability to hold gaze stable also declines more quickly outside 20° (Bertolini et al., 2013). In daily lives, when we are free to move our heads, a shift of gaze larger than 20° is commonly accompanied by a head movement. Given the intense coupling between attention and saccades (Sheliga et al., 1994; Hoffman and Subramaniam, 1995), it is reasonable to speculate that attentional processing may change quite differently inside vs. outside 20° of eccentricity. Therefore, research on pre-cuing effects of spatial attention needs to expand more into the periphery.

There are two types of pre-cues influencing spatial attentional processing. An exogenous pre-cue occurs at a peripheral location and automatically attracts attention to the location; whereas an endogenous pre-cue appears centrally and indicates where attention should be allocated to. Although both exogenous and endogenous pre-cues affect early visual processing (e.g., Brefczynski and DeYoe, 1999; Gandhi et al., 1999; Pinsk et al., 2004), much evidence points to differential mechanisms of the two. Impacts from an exogenous pre-cue is stimulus-driven, involuntary, quick and transient; in contrast, impacts from an endogenous pre-cue is concept-driven, voluntary, slower but more sustained (e.g., Posner, 1980; Jonides, 1981; Nakayama and Mackeben, 1989; Ling and Carrasco, 2006). Attentional shifts associated with an exogenous pre-cue depends minimally on the distance; however, shifts associated with an endogenous pre-cue is significantly affected by distance (Chakravarthi and VanRullen, 2011). Compare to exogenous pre-cueing, endogenous pre-cueing involves more cognitive control, and could be a potential mechanism for cognitive penetration of perception. For example, attentional shifts to spatial locations implied by an endogenous cue depend on the validity of the cue (Sperling and Melchner, 1978; Mangun and Hillyard, 1990; Giordano et al., 2009). In addition, several recent studies have demonstrated that probabilities of targets’ occurrence in various areas of the visual field could be learnt and guide the distribution of attention (Geng and Behrmann, 2002; Druker and Anderson, 2010). On the contrary, an exogenous pre-cue automatically attracts attention even when the cue is uninformative (Pestilli et al., 2007; Montagna et al., 2009; Yeshurun and Rashal, 2010). Previous studies that examined eccentricity effects on pre-cueing have dominantly used exogenous pre-cues. Therefore, investigation of endogenous pre-cues with the target occurring across a wide range of eccentricities (particularly beyond 20°) is needed. Such exploration will provide valuable evidence for cognitive penetrability of visual perceptual processing (for a review, see Lupyan, 2015), and particularly on the impacts from endogenous spatial attention on early vision across a large area of the visual field.

In the present study, we used a task measuring the spatial distribution of attention across an extended area of the visual field (Attentional Visual Field Task; Spence et al., 2013; Feng and Spence, 2014). On this task, the distribution of attention across a large area of the visual field can be reflected by performance in target detection which decreases with the increase in target eccentricity (e.g., Feng and Spence, 2014; Feng et al., 2016). In the current study, we implemented endogenous cues that occurred before the stimulus displays, to examine the pre-cueing effects across a wide range of visual eccentricities (10°, 20°, and 30°). To ensure that we were measuring early visual processing (within 120 ms after stimulus presentation; Raftopoulos, 2009; Raftopoulos and Zeimbekis, 2015), the stimuli were displayed very briefly and followed by a mask in the experiments (20 or 30 ms). Our study also focused on covert attention that is the orienting of attention without an eye movement, although covert attention usually contribute to a subsequent eye movement to the attended location (Peterson et al., 2004). In the experiments, we designed the tasks in which either an eye movement would not be very useful (Experiment 1 on modifying the size of the to-be-attended area), or the time interval between the onsets of a pre-cue and a target was too brief to allow the execution of an eye movement (which normally takes at least 200 ms; Johnson and Proctor, 2004). In this study, we investigated two types of endogenous pre-cues that provide location information of a target: (1) a pre-cue that indicated the size of the area in which a target is likely to occur (Experiment 1), and (2) a pre-cue that showed the direction of a target in the visual field (Experiment 2).

## Experiment 1

Experiments 1A and 1B used cues that indicated the size of the area in which a target was likely to occur. Participants were instructed to make use of the cue and respond both accurately and quickly on the Attentional Visual Field (AVF) task. The experimental procedures were approved by the University of Toronto Ethics Review Board.

### Experiment 1A

A cue was presented only once, at the beginning of each block of trials. Therefore, the size of the area indicated by the cue was identical through the entire block of trials. Because the expectation of the size of the area was formed at the beginning of each block, the participant did not need to adjust the expectation on every trial, thus minimizing the cognitive overhead.

#### Methods

##### Participants

Fifteen undergraduates at the University of Toronto (six males, nine females; age range: 18–22 years), participated for course credit. All participants reported normal or corrected-to-normal vision.

##### Stimuli

**FIGURE 1:**
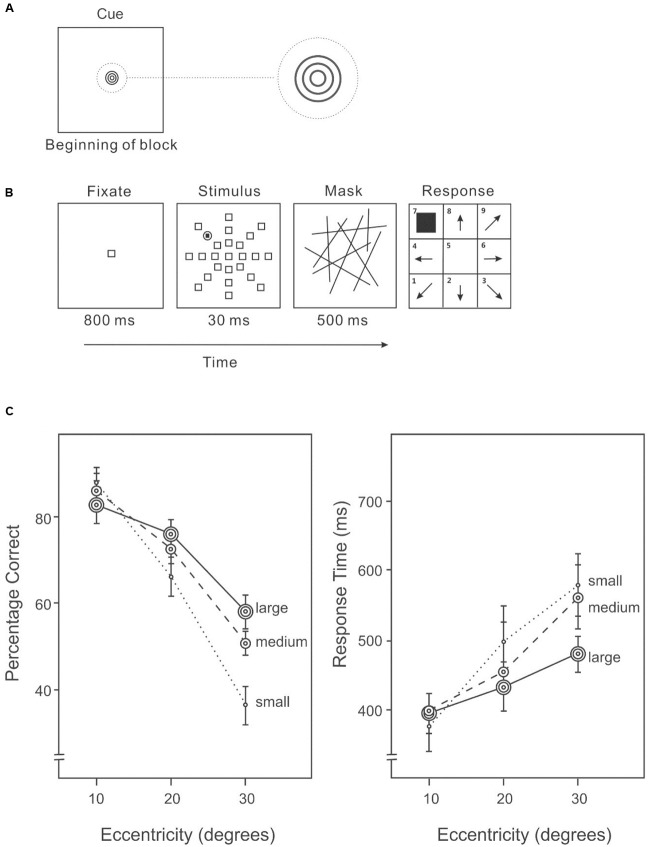
**(A)** A cue indicating the size of the area in which the target was likely to appear was given before each block of trials in Experiment 1A. **(B)** A sample trial of the Attentional Visual Field (AVF) task. **(C)** Percentage correct (left panel) and response time (right panel) on the AVF task with an area size cue.

An AVF task was used to examine the distribution of attention. Before each block of trials, a cue indicating the likely size of the area containing the target was presented (**Figure 1A**). The cue to a small area was a dark-gray unfilled circle (2.2° × 2.2°); to a medium area, the cue was two dark-gray concentric unfilled circles (3.6° × 3.6°); and to a large area the cue was three dark-gray concentric unfilled circles (4.5° × 4.5°). In each trial of the AVF task (**Figure 1B**), the stimuli were presented in a circular area (63.1° diameter) centered on a uniform light-gray screen. Each trial began with a centered, unfilled fixation square with a dark-gray border (3° × 3°) presented for 800 ms. The stimulus display consisted of 23 identical distractors and one target, each uniquely localized at an eccentricity of 10°, 20°, or 30° in one of eight equally spaced directions. The location of the target was randomly selected on each trial, subject to the restriction that the target appeared an equal number of times in each possible location over the block of trials. The target was a dark-gray filled square (1.5° × 1.5°) surrounded by an unfilled circle with a dark-gray circumference (3° × 3°). The distractors squares were unfilled squares with dark-gray borders (3° × 3°), identical to the fixation square. The stimulus display was presented for 30 ms, followed by a mask of randomly oriented dark-gray lines for 200 ms. Participants indicated the direction of the target after the mask disappeared. The next trial started 1000 ms after a response was made.

##### Design

The experimental design was a completely within-participant 3 × 3 repeated-measures design. Cued area size (small/medium/large) was a block factor, and target eccentricity (10°/20°/30°) was varied within each block. There were three blocks for each cued area size and the order presented in a counterbalanced order.

##### Procedure

Before the experiment, the meanings of the size cues were explained to participants: a small size cue (only one small circle) indicated that the target was likely to occur only at an eccentricity of 10°; a medium size cue (two concentric circles) indicated that the target was likely to occur at an eccentricity of either 10° or 20°; and a large size cue (three concentric circles) indicated that the target was likely to occur at any eccentricity: 10°, 20°, or 30°. Participants positioned their head on a chin rest at a distance of 35 cm from the display. The AVF task was programed in Microsoft Visual Studio C++ and administered on a PC for experiment in the lab. A practice session, consisting 36 trials was required to ensure that participants understood the task. The 36 practice trials were grouped into three blocks: 12 trials for each area size cue. In the experimental session, trials with the same size cue were blocked and repeated three times, for a total of nine blocks that were counterbalanced using Latin Square. There were 72 trials in each block with a large size cue. Because a large cue would be valid for a target appearing at any of the three eccentricities, every trial with a large size cue was valid. The number of trials in the block, 72, was a multiple of the 24 possible locations of the target. In contrast, there were 80 trials in each block with a medium or small cue because there were both valid and invalid trials for these cues (80% cue validity). A small or medium size cue would be invalid for a target appearing at an eccentricity outside the to-be-attended area (an eccentricity of 20° or 30° with a small size cue or an eccentricity of 30° with a medium size cue); therefore, blocks with medium or small size cues had both valid and invalid trials. Participants saw the size cue before each block and were asked to maintain the same expectation induced by this cue throughout the block. Participants were given a 2-min rest after each block. Responses from participants indicating the directions of the target on each trial and the response times were recorded.

#### Results

A 3 × 3 (cued size: small/medium/large, target eccentricity: 10°/20°/30°) repeated-measures ANOVA was used to analyze the percentage of correct responses and response time data. We calculated the percentage of correct responses and average response time based on all trials of each combination of conditions.

##### Percentage correct

Overall accuracy on target detection differed significantly among eccentricities (10°: 81%, 20°: 68%, 30°: 48%) (**Figure 1C**, left panel), *F*(2,28) = 60.19, *p* < 0.001. In particular, accuracy was higher at 10° than 20°, *F*(1,14) = 34.02, *p* < 0.001, and also higher at an eccentricity of 20° than 30°, *F*(1,14) = 51.75, *p* < 0.001. Overall accuracy varied with cue size (small cue: 62%, medium cue: 67%, large cue: 68%), *F*(2,28) = 7.05, *p* < 0.01. Subsequent analyses revealed a significantly difference between a small cue and a medium cue, *F*(1,14) = 12.95, *p* < 0.01, and between a small and a large cue, *F*(1,14) = 9.31, *p* < 0.01. There was a significant interaction between expected size and target eccentricity, *F*(2,86) = 15.41, *p* < 0.01. In particular, accuracy differed significantly among cued size at an eccentricity of 30°, *F*(2,28) = 14.79, *p* < 0.001, and at an eccentricity of 20°, *F*(2,28) = 3.77, *p* < 0.05, but not at an eccentricity of 10°, *F*(2,28) = 2.39, *p* = 0.11.

##### Response time

Response speed differed among eccentricities of the target (**Figure 1C**, right panel), *F*(2,28) = 6.27, *p* < 0.01. The interaction between cued size and target eccentricity was significant, *F*(4,56) = 2.68, *p* < 0.05. Slower responses were associated with lower accuracies (**Figure 1C**), suggesting that there was no speed-accuracy trade-off.

### Experiment 1B

Experiment 1A demonstrated that the attended area could be modified by a cue that indicated the likely eccentricity of the target. This experiment examined whether a cue that varied unpredictably would still be effective when presented before each trial rather than before the block of trials. Thus the time available to make use of the cue was much shorter. Otherwise, the task was identical to that in Experiment 1A. Since changing the size of the attended area takes processing time (Eriksen and St. James, 1986), the influence of the cue may not be as large as in Experiment 1A.

#### Methods

##### Participants

Fifteen undergraduates at the University of Toronto (5 males, 10 females; age range: 17–22 years) participated for course credit. All participants reported normal or corrected-to-normal vision.

##### Task

**FIGURE 2:**
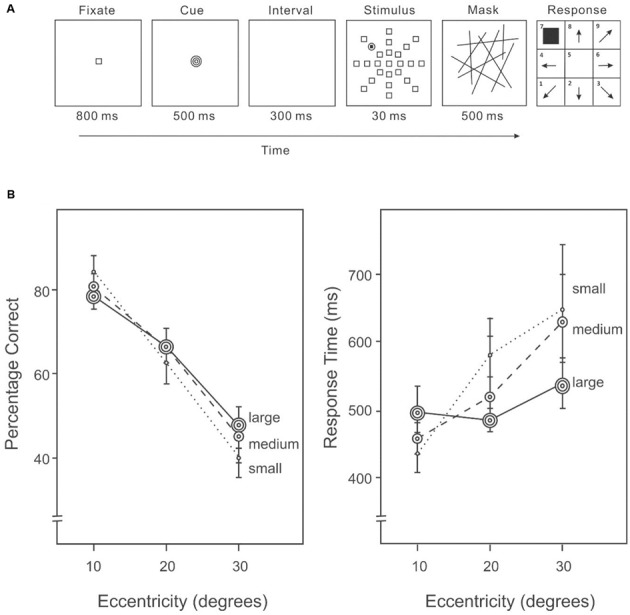
**(A)** A sample trial of the AVF task with an area size cue in Experiment 1B. An endogenous cue indicating the size of the area in which the target was likely to appear was given immediately following the fixation in each trial. **(B)** Percentage correct (left panel) and response time (right panel) on the AVF task with an area size cue.

All settings were the same as in Experiment 1A, except the cue was presented after the fixation and before the stimulus on each trial (**Figure 2A**). Each cue was presented for 500 ms and followed by a 300 ms interval, before the onset of the stimulus display.

##### Design

This experiment adopted a completely within-participant 3 × 3 repeated-measures design. Factors include cued area size (small/medium/large) and target eccentricity (10°/20°/30°). Trials of combinations of cued area size and target eccentricity were mixed and randomized.

##### Procedure

These were the same as in Experiment 1A, except the order of the trials was randomized. There were 36 randomized practice trials. In the experimental session, 720 trials were presented in random sequence with the target appearing 30 times in each of the 24 locations. Overall, 80% of the trials were valid, in which the cued size was *equal to or larger than* the eccentricity of presented target (cue validity was 100% for a large size cue, 88% for a medium size cue, and 53% for a small size cue). Participants were given a 2-min rest after each block of 120 trials. Responses from participants indicating the directions of the target on each trial and the response time were recorded.

#### Results

A 3 × 3 (cued size: small/medium/large, target eccentricity: 10°/20°/30°) repeated-measures ANOVA was used to analyze the percentage of correct responses and response time data. We calculated the percentage of correct responses and average response time based on all trials of each combination of conditions.

##### Percentage correct

Target detection differed among eccentricities (10°: 82%, 20°: 70%, 30°: 49%) (**Figure 2B**, left panel), *F*(2,28) = 50.34, *p* < 0.01. Subsequent contrasts revealed that accuracy was higher at 10° than 20°, *F*(1,14) = 13.70, *p* < 0.001, and also higher at an eccentricity of 20° than 30°, *F*(1,14) = 69.19, *p* < 0.001. Varying the size of the cued area did not change overall performance (small cue: 66%, medium cue: 68%, large cue: 68%), *F*(2,28) = 0.40, *p* = 0.67; however, the interaction between cue and eccentricity was significant (**Figure 2B**, left panel), indicating that the distribution of attention was modified according to the cued area size, *F*(4,56) = 5.34, *p* < 0.01.

##### Response time

Response speed was different among eccentricities (10°: 429 ms, 20°: 484 ms, 30°: 580 ms) (**Figure 2B**, right panel), *F*(2,28) = 8.68, *p* = 0.001. The interaction between cue and eccentricity was also significant, *F*(4,56) = 4.68, *p* < 0.01. Slower responding was associated with lower accuracy (**Figure 2B**), suggesting that there was no speed-accuracy trade-off.

Given the pre-cueing effect was more visible in Experiments 1A than 1B, we conducted a statistical comparison of the pre-cueing effects in Experiments 1A and 1B using a 3 × 3 × 2 repeated-measure ANOVA on both accuracy and response time. Within-subject factors include cued area size (small/medium/large) and target eccentricity (10°/20°/30°). Between-subject factor was cue style (blocked/mixed). There was a marginally significant interaction between cued area size and cue style on accuracy, *F*(2,56) = 3.06, *p* = 0.05, but not response time, *F*(2,56) = 0.50, *p* = 0.61.

We also investigated the difficulty of increasing, decreasing or keeping the to-be-attended area constant from one trial to the next using a single-factor (area change, three conditions: increasing, unchanging, and decreasing) repeated-measures ANOVA. To allow opportunities to increase, decrease, and unchanged the cue size, only valid trials with medium size cues were included in this analysis (e.g., a trial with a large size cue would not be a result of a decrease in the attended area). A trial (with a medium size cue) preceded by a trial with a large cue was included in the decreasing condition: participants had to reduce the size of the to-be-attended area from a large size in the previous trial to a medium size in the current trial. Similarly, a trial preceded by a trial with a medium cue was included in the unchanging condition, and a trial preceded by a trial with a small cue was included in the increasing condition. The percentages of correct among the three conditions were comparable (decreasing: 74%, unchanging: 74%, increasing: 76%), *F*(2,28) = 0.97, *p* = 0.39. In terms of response time, there was a trend of differential response speeds among the three conditions (decreasing: 551 ms, unchanging: 504 ms, increasing: 524 ms), *F*(2,28) = 3.08, *p* = 0.06. Compared to the unchanging condition, response was much slower when participants had to decrease the to-be-attended area, *F*(1,14) = 7.49, *p* < 0.05, but comparable when they had to increase the area, *F*(1,14) = 0.73, *p* = 0.41.

### Discussion

Our findings suggest that expectation modified the size of the attended area and hence attentional processing at eccentricities of 20° and 30°. These findings were in line with Titchener (1908), Eriksen and St. James (1986) that the distribution of attention can be modified, according to the participant’s expectation. For example, when highly focused on a primary central task, participants were less capable of noticing stimuli presented outside the area of the primary task (Ikeda and Takeuchi, 1975; Williams, 1995). Using the same size cue throughout a block of trials (Experiment 1A) was more effective (marginally) in modifying the distribution of attention than presenting a potentially different size cue on each trial (Experiment 1B). This is at variance with other experiments that used blocked trials (similar procedure to Experiment 1A), where no differences were observed as the result of manipulations of the spatial cues (Posner, 1978; Remington and Pierce, 1984). However, a similar experiment to Experiment 1B with randomized trials did show differences (Downing and Pinker, 1985). These discrepancies may lie in the nature of the tasks used in the studies. One possible reason is that in our experiment 1B, requiring participants to use a potentially different size cue on each trial must inevitably increase the cognitive workload and thus it is unsurprising that performance should suffer relative to the blocked trials of Experiment 1A. Another explanation is that a frequent short-term repetition of target locations may lead to more significant cueing effects from learnt statistics. Walthew and Gilchrist (2006) found that while participants learnt and used statistics of the target location to guide their detection, the benefits of such endogenous cue were eliminated when short-term repetitions of target locations were restricted. It may be the case that in our Experiment 1, the repetitions of target locations were higher with the blocked design (Experiment 1A) than with the randomized trial design (Experiment 1B). But it is also interesting to consider our finding that the cueing benefits were greater at larger eccentricities despite the fact that the highest repetition would have occurred at the small size cue block (i.e., when the target frequently occurred at the eccentricity of 10°). It is important to note that another study found that participants were capable of learning relatively complex statistical patterns (Druker and Anderson, 2010). Therefore, more complex patterns over trials may have also played a role in influencing our participants’ allocation of attention in our experiment.

## Experiment 2

We examined the influence of a cue indicating the likely direction of the target. This directional cue was highly valid (67% in Experiment 2A and 80% in Experiment 2B) to encourage participants to use the cue (Jonides, 1981; Kröse and Julesz, 1989; Wright and Ward, 2008). The validities were convenient choices based on the number of conditions and repetitions in each experiment. Participants reported the direction (Experiment 2A) or identity (Experiment 2B) of the target. During covert orienting of attention, only the attentional focus but not the fixation is shifted. To help ensure that the participant maintained fixation during each trial, the duration from onset of the cue to offset of the stimulus was limited to 200 ms. Thus the likelihood of a saccade occurring during processing of the stimulus was low (Sparks et al., 2000). The experimental procedures were approved by the University of Toronto Ethics Review Board.

### Experiment 2A

An arrow (67% valid) indicating the likely direction of the target was presented before the AVF stimulus appeared. Participants reported the direction of the target.

#### Methods

##### Participants

Fourteen undergraduates at the University of Toronto (five males, nine females; age range: 18–21 years), participated for course credit. All participants reported normal or corrected-to-normal vision.

##### Task

**FIGURE 3:**
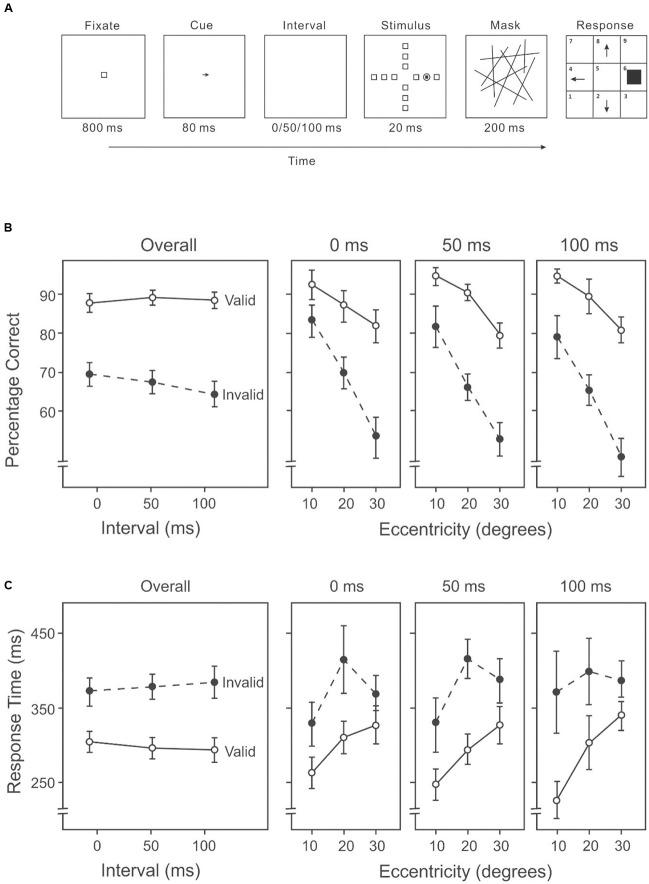
**(A)** A sample trial of the AVF task with an endogenous cue indicating the likely direction of the target in Experiment 2A. **(B)** Percentage correct on the attention orienting AVF task, including overall accuracies across intervals (left panel) and accuracies at each interval across eccentricities (three right panels). **(C)** Response time on the attention orienting AVF task, including overall response times across intervals (left panel) and response times at each interval across eccentricities (three right panels).

This experiment used the AVF task with an endogenous cue (an arrow) indicating the likely direction of the target between the fixation and the stimulus display (**Figure 3A**).

##### Design

We used a 3 × 3 × 2 within-subject repeated-measures design, whose factors were target eccentricity (10°/20°/30°), cue-target interval (0/50/100 ms) and target validity (valid/invalid).

##### Stimuli

The AVF task was very similar to the one used in Experiment 1B, except for the number of distractors, the cue, and the exposure settings. In this experiment, the directional cue, a dark-gray arrow (3° × 3°), was presented at the center of the screen. The cue pointed in one of four directions (up, down, left, or right). During each trial, the cue remained on screen for 80 ms and was followed by a blank display (0, 50, or 100 ms) and then the stimulus, which consisted of 11 identical distractors and one target, each uniquely localized at an eccentricity of 10°, 20°, or 30° in one of four equally spaced directions (up, down, left, or right). The location of the target was randomly selected on each trial, subject to the restriction that the target appeared an equal number of times in each possible location. The stimulus display was presented for 20 ms, followed by a mask of randomly oriented lines for 200 ms. Participants indicated the direction of the target after the mask disappeared. The next trial started 800 ms after a response was made.

##### Procedure

Participants were required to position their head on a chin rest at a distance of 35 cm from the screen. A practice session of 24 trials was used to ensure that participants understood the task. In the experimental session, 648 trials were presented in a random sequence with the target appearing 54 times in each of the 12 locations for each cue-target interval condition. Sixty-seven percentage of the trials were valid (the cue arrow correctly pointed to the target). Participants were informed that the cue was valid in 67% of the trials, to encourage them to use the cue. In the invalid trials, the cue arrow pointed in one of the other three directions, at random with equal frequency. Participants were given a 2-min rest after each set of 108 trials. Responses from participants indicating the directions of the target and the reaction times on each trial were recorded.

#### Results

A 3 × 3 × 2 (target eccentricity: 10°/20°/30°, cue-target interval: 0/50/100 ms, cue validity: valid/invalid) repeated-measures ANOVA was used to analyze the percentage of correct responses and response time data. We calculated the percentage of correct responses and average response time based on all trials of each combination of conditions.

##### Percentage correct

Target detection differed significantly among the three eccentricities (10°: 87%, 20°: 78%, 30°: 65%) (**Figure 2B**, three right panels), *F*(2,26) = 55.45, *p* < 0.001. Subsequent contrasts suggested that accuracy in target detection was higher at an eccentricity of 10° than 20°, *F*(1,13) = 74.08, *p* < 0.001, and also higher at an eccentricity of 20° than 30°, *F*(1,13) = 35.11, *p* < 0.001. The mean accuracy on the valid trials was higher than that on the invalid trials (valid: 87%, invalid: 66%) (**Figure 3B**), *F*(1,13) = 70.60, *p* < 0.001. The duration of the cue-target interval also had an impact on the performance. With varying intervals, overall accuracy (including both valid and invalid trials) differed significantly (0 ms: 77%, 50 ms: 77%, 100 ms: 75%), *F*(2,26) = 3.48, *p* < 0.05. Subsequent contrasts among individual conditions revealed a significant difference between 0 and 100 ms, *F*(1,13) = 9.87, *p* < 0.01. There was a significant interaction between validity and cue-target interval, *F*(2,26) = 3.91, *p* < 0.05. Subsequent analyses showed that accuracy on invalid trials varied significantly among intervals (0 ms: 68%, 50 ms: 66%, 100 ms: 63%) (**Figure 2B**, left panel), *F*(2,26) = 6.59, *p* < 0.01; whereas accuracy on valid trials did not differ among intervals (0 ms: 87%, 50 ms: 88%, 100 ms: 87%) (**Figure 3B**, left panel), *F*(2,26) = 0.14, *p* = 0.87. There was also a significant interaction between cue validity and target eccentricity, *F*(2,26) = 8.46, *p* = 0.001 (**Figure 3B**, three right panels). No other two-way or three-way interaction was significant.

##### Response time

Response speed differed among eccentricities (10°: 297 ms, 20°: 358 ms, 30°: 358 ms) (**Figure 3C**, three right panels), *F*(2,26) = 6.71, *p* < 0.01. Subsequent contrasts revealed faster response speed at 10° than 20°, *F*(1,13) = 25.01, *p* < 0.001, and faster speed at 10° than 30°, *F*(1,13) = 9.93, *p* < 0.01. Participants also responded faster in valid trials than in invalid trials (valid: 297 ms, invalid: 379 ms) (**Figure 3C**), *F*(1,13) = 47.91, *p* < 0.001. But the duration of the cue-target interval had no effect on response time (0 ms: 338 ms, 50 ms: 337 ms, 100 ms: 339 ms) (**Figure 3C**, left panel), *F*(2,26) = 0.01, *p* = 0.99. Overall, higher accuracy was associated with shorter RTs (**Figures 3B,C**), suggesting that there was no speed-accuracy trade-off.

### Experiment 2B

Findings from Experiment 2A showed that the directional cue affected participants’ performance on the AVF task. However, given that participants responded with the direction of the target, there may be a guessing bias as the cue on target direction was highly valid. When participants were not sure about the direction of the target, they may have responded with the cued direction. To eliminate the possible influence of guessing bias in the effect of directional cueing, we changed the task from reporting the direction of the target to reporting the identity of the target in Experiment 2B. The target in the AVF task was one of two visually distinct objects and participants reported which object had been presented. Because there was no relationship between the identity of the target and the cued direction, participants were not able to improve performance by guessing.

#### Methods

##### Participants

Twenty undergraduates at the University of Toronto (8 males, 12 females; age range: 17–23 years) participated for course credit. All participants reported normal or corrected-to-normal vision.

##### Task

**FIGURE 4:**
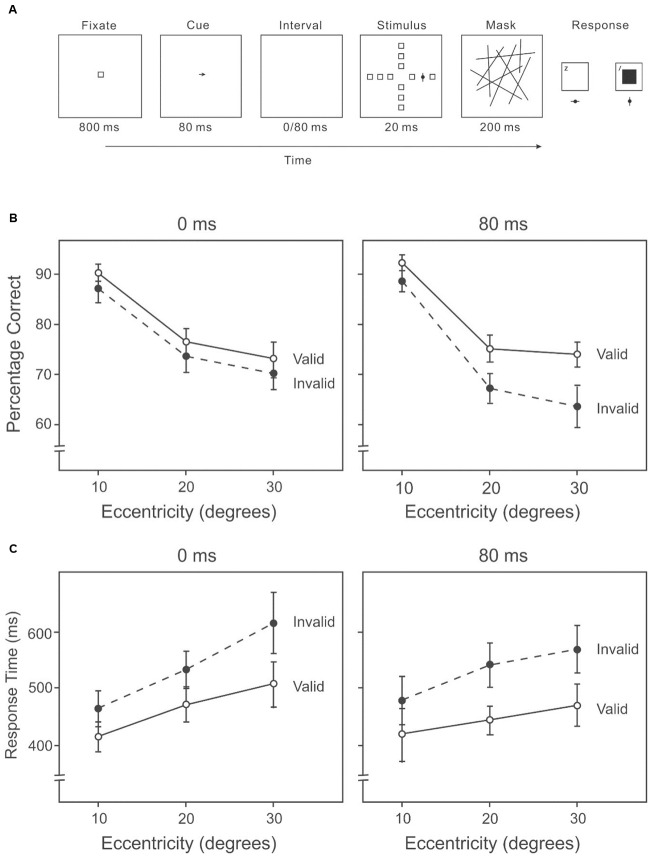
**(A)** A sample trial of the AVF identification task with an endogenous cue indicating the direction of the target in Experiment 2B. Participants reported the identity of the target. **(B)** Percentage correct on the attention orienting AVF identification task at each interval across eccentricities. **(C)** Response time on the attention orienting AVF identification task at each interval across eccentricities.

The task was very similar to Experiment 2A except participants reported the identity of the target (the target was now bisected by either a horizontal or a vertical line) rather than the direction of the target (**Figure 4A**). Only two cue-target intervals (0/80 ms) were used to limit the number of trials.

##### Design

We used a 3 × 2 × 2 within-subject repeated-measures design, whose factors were target eccentricity (10°/20°/30°), cue-target interval (0/80 ms) and target validity (valid/invalid).

##### Stimuli

All task settings were the same as in Experiment 2A, except the target was a filled dark-gray circle (2.2° × 2.2°) with a dark-gray line (0.8° × 3.6°) bisecting the circle either horizontally or vertically. After a blank interval of 0 or 80 ms, the stimulus display appeared for 40 ms, followed by a mask of 200 ms duration. Participants responded after the mask disappeared.

##### Procedure

As in Experiment 2A, except that participants reported the identity of the target, pressing ‘Z’ for targets with the horizontal line and ‘/’ for targets with the vertical line. Participants pressed ‘Z’ with the left hand, and ‘/’ with the right hand. Participants were instructed to respond both accurately and quickly. The choices of target identity and the reaction times were recorded. After a practice session of 24 trials, participants completed 720 trials in a random sequence with the target appearing 30 times in each of the 12 locations, for each of the two cue-target interval conditions. Eighty percentage of the trials were valid.

#### Results

A 3 × 2 × 2 (target eccentricity: 10°/20°/30°, cue-target interval: 0/80 ms, cue validity: valid/invalid) repeated-measures ANOVA was used to analyze the percentage of correct responses and response time data. We calculated the percentage of correct responses and average response time based on all trials of each combination of conditions.

##### Percentage correct

Accuracy in target detection varied among eccentricities (10°: 87%, 20°: 71%, 30°: 69%) (**Figure 4B**), *F*(2,38) = 68.53, *p* < 0.001. Subsequent contrasts revealed higher accuracy at 10° than 20°, *F*(1,19) = 111.37, *p* < 0.001, and higher accuracy at 10° than 30°, *F*(1,19) = 78.17, *p* < 0.001. Accuracy was higher on valid trials than invalid trials (valid: 77%, invalid: 74%), *F*(1,19) = 10.20, *p* < 0.01. There was a significant interaction between cue validity and cue-target interval, *F*(1,19) = 10.06, *p* < 0.01. Subsequent analyses showed that when the cue-target interval was 0 ms, there was little difference in accuracy between valid and invalid conditions (valid: 77%, invalid: 76%) (**Figure 4B**, left panel), *F*(1,19) = 1.40, *p* = 0.25. However, with an interval of 80 ms, the difference was significant (valid: 78%, invalid: 72%) (**Figure 4B**, right panel), *F*(1,19) = 18.88, *p* < 0.01. No other two-way or three-way interaction was significant.

##### Response time

Response speed was different among eccentricities (10°: 449 ms, 20°: 494 ms, 30°: 545 ms) (**Figure 4C**), *F*(2,38) = 16.62, *p* < 0.01. Response was much faster at 10° than 20°, *F*(1,19) = 22.60, *p* < 0.001, and faster at 20° than 30°, *F*(1,19) = 19.81, *p* < 0.001. Participants responded faster with valid cues than with invalid cues (valid: 463 ms, invalid: 529 ms), *F*(1,19) = 44.91, *p* < 0.01. When the cue-target interval was zero, the mean reaction time was shorter with valid cues than with invalid cues (valid: 476 ms, invalid: 534 ms) (**Figure 4C**, left panel), *F*(1,19) = 20.41, *p* < 0.01. With an interval of 80 ms, participants responded faster with valid cues than with invalid cues (valid: 450 ms, invalid: 525 ms) (**Figure 4C**, right panel), *F*(1,19) = 34.39, *p* < 0.01. Higher accuracies were generally accompanied by faster response times (**Figures 4B,C**), suggesting that there was no speed-accuracy trade-off.

### Discussion

Experiments 2A and 2B demonstrated that expectation of the direction of the target can change the distribution of attention. An increase of the cue-target interval produced a more pronounced effect with endogenous covert orienting of attention. This is evident in both Experiments 2A and 2B. Particularly, in Experiment 2B, as the cue-target interval increased from 0 to 80 ms, participants’ percentage of correct responses became significantly higher when the target appeared in the expected direction (valid conditions) compared to when the target appeared in unexpected directions (invalid conditions). Presumably, with a longer cue-target interval, participants had more time to form their expectation of the likely direction (Posner, 1980; Shepherd and Müller, 1989). Thus the difference in accuracy between the valid and invalid conditions became greater. In both Experiments 2A and 2B, this greater difference with a prolonged interval was caused by further impairment in accuracy by an invalid cue, rather than by an enhancement in accuracy by a valid cue. This may imply that, when discrimination and identification of a target among distractors are necessary, expectation of the direction of the target improves performance mostly by inhibiting the unexpected directions between 80 and 180 ms (160 ms in Experiment 2B) following the onset of the cue. When the interval was 0 ms, the stimuli display appeared immediately after the cue display. This may have led to a masking effect. However, given the cue only occurred at the center while the target and distractors occurred at eccentricities of 10°, 20°, and 30°. There should be minimal interference between the cue and target displays.

## General Discussion

Two forms of expectations induced by spatial cues on attentional distribution were examined in the presented experiments. As predicted, the results suggested that a participant can control the size of the area in which attention is deployed, and can covertly orient attention in a particular direction. Modification of the distribution of attention is an efficient mechanism for enhancing attentional performance when the cue is valid. The cues used in the experiments were endogenous (i.e., directed participants’ attention to the spatial location of the target). Endogenous covert distribution of attention induced by an expectation of the target is an efficient mechanism for enhancing attentional performance when the prediction is highly accurate. We measured covert attention that reflected the effect of pre-cueing attention without an eye movement. Our experiments were designed to eliminate voluntary eye movements that could benefit task performance based on the cue. In Experiment 1, although the interval between the onsets of a cue and a target was long (e.g., 800 ms in Experiment 1B), an eye movement would not have been beneficial as a target could occur at any direction within the visual area. In Experiment 2, although an eye movement toward the cued direction could be beneficial, the cue-target interval was too brief for an eye movement to be executed. A significant advantage of endogenous covert distribution of attention across the extended attentional visual field is that detection of a target that appears some distance from fixation is faster. Endogenously orienting attention to a new location can be faster than making an eye movement (Johnson and Proctor, 2004), and usually contribute to a subsequent eye movement to the attended location (Peterson et al., 2004).

We found that with a larger size of the cued area, overall accuracy of target detection was lower, implying a reduction of the average attentional intensity within the attended area. This inverse relationship between the size of the attended area and the attentional intensity (Feng and Spence, 2013, p.154) was first documented by Wolff (1738; 1740, translated and interpreted in Hatfield, 1998) in his *Psychologia Empirica* (1738) and *Psychologia Rationalis* (1740). Later, similar ideas were implied in Titchener’s (1908) Law of Two Levels and also the zoom-lens model of selective attention (Eriksen and Yeh, 1985; Eriksen and St. James, 1986). In the zoom-lens model, it is assumed that attention is distributed evenly across the selected area except there is a gradual decrease of the attentional intensity near the boundary (Eriksen and St. James, 1986); whereas performance on the AVF task suggests that the default (uncued) distribution of attention is more like a unimodal probability distribution, with lower attentional intensity at locations further from fixation. Experiment 1 also suggested that it was more difficult to modify than to maintain the size of the to-be-attended area. This increase in difficulty was particularly profound when the size of the to-be-attended area had to be reduced. Future explorations on potential underlying neural mechanisms are necessary.

Our results suggested that modifying the size of the attended area takes time to complete. This is evident in both Experiments 2A and 2B. In Experiment 2A, the advantage on valid trials, compared to invalid trials, was greater with a longer cue-target interval (comparing among intervals of 0, 50, and 100 ms). In Experiment 2B, the advantage on valid trials increased considerably when the interval was increased from 0 to 80 ms (from a non-significant difference in accuracy and RT, to a much higher accuracy and faster RT on valid trials). Improved performance with a valid endogenous cue and a longer cue-target interval has been demonstrated at eccentricities of 10° and 20° (Shepherd and Müller, 1989). But in Shepherd and Müller (1989) study, the target was presented without distractors, thus only target detection (no discrimination or identification) was necessary. Experiments 2A and 2B suggested that this effect holds when discrimination, localization, and identification (finding the target in the presence of distractors) were also involved. And the effect holds not only at eccentricities of 10° and 20°, but also at an even more extreme eccentricity of 30°. Moreover, the benefit from a valid cue is progressively greater at locations further from fixation. Furthermore, in both Experiments 2A and 2B, with a longer cue-target interval, a valid cue did not further facilitate identification of the target, but an invalid cue further impaired the identification. This differs from the findings in Shepherd and Müller (1989). In Shepherd and Müller (1989), with a longer time following the onset of the cue (increased from 50 to 150 ms), the accuracy on target detection was further enhanced with a valid cue, and further impaired with an invalid cue. Notably, in Shepherd and Müller (1989), there was no distractor presented together with the target; in contrast, in Experiments 2A and 2B, the stimuli consisted of a target and eleven distractors. This may imply that, when only detection is involved, both enhancement in the expected direction and inhibition in the unexpected directions occur (between 50 and 150 ms following the onset of the cue). However, if discrimination and identification are also necessary, inhibition in the unexpected directions may have played a major role (between 80 and 180 ms following the onset of the cue).

### A Conceptual Model of Spatial Attention across the Visual Field

**FIGURE 5:**
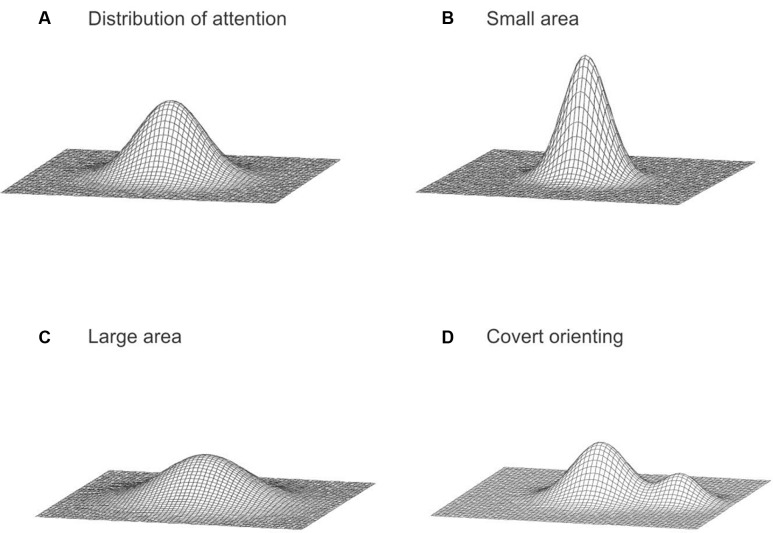
**(A)** The distribution of attention across the visual field. **(B)** A concentrated distribution with a small attended area. **(C)** A distribution with a large attended area. **(D)** A possible bimodal distribution resulting from endogenous covert orienting of attention.

Based on our findings, we conceptualize the spatial distribution of attention as a bivariate probability distribution over the visual field (**Figure 5A**; Feng, 2011), similar to the idea describing the distribution of attention as a gradient of the attentional resource around the focus of attention (Baldwin, 1889; Downing and Pinker, 1985; Eriksen and Yeh, 1985; LaBerge and Brown, 1989; James, 1890; Palmer, 1990; Müller et al., 2005). Our model specifically considers a large area of the visual field. The probability density at any particular location in the visual field represents the attentional intensity corresponding to that location (Feng and Spence, 2013, p.154). In general, the attentional intensity decreases with an increase in the distance from the fixation (Feng and Spence, 2014; Feng et al., 2016). Influences from expectations induced by pre-cueing were assumed to modify the distribution of attention. When a participant expects a target to appear anywhere within a large area, the spread of the distribution of attention is larger, to accommodate the greater uncertainty (**Figures 5B,C**), thus attentional processing in the periphery is increased. Consequently, since attention is assumed to be a fixed resource, the distribution flattens as it spreads. However, when a participant expects a target to appear in a particular direction, the distribution of attention is gradually shifted in that direction (**Figure 5D**), resulting in an increase in attentional processing of information at the expected direction. Our earlier work also suggests that there are pre-existing biases in the attentional distribution (Feng and Spence, 2014; Feng et al., 2016). However, it is important to note that the simple model proposed in this paper is rudimentary and was intended to provide a qualitative description. Elaboration of the model could specify particular probability distributions and how these may be modified and further examination is necessary to further specify the model with more details. It is also critical to point out that this descriptive model only intends to describe possible spatial mechanisms of attention, which is only one aspect of the operation of attention. There are many non-spatial processes by attention (e.g., Egly et al., 1994; Moore et al., 1998; Lupyan, 2008; Lupyan and Spivey, 2010; Baldauf and Desimone, 2014). For example, when the image of a face and a house superimposed (thus the two objects are at the same spatial location), we can choose to pay attention to either one and ignore the other (Baldauf and Desimone, 2014). Another example is cross-modal facilitation given category-based attention (Lupyan and Spivey, 2010). Our visual processing of an item is enhanced when we hear a similar label. The proposed descriptive model is limited to the spatial processing of attention; it does not capture these non-spatial attentional processing, nor it explains potential interactions between space-based and object-based mechanisms.

### Implication of Eccentricity Effect on Cognitive Penetration of Perception

In the traditional views, attention has been conceptualized as a passive filter or gate-keeper (Broadbent, 1958; Posner, 1980) that selectively facilitates the processing of some information (e.g., targets) while inhibiting others (e.g., distractors). More recent views challenge this conceptualization and propose attention’s role in active construction of perceptual representation under the influence from cognition (e.g., Lupyan, 2015; Nanay and Fazekas, 2017). This proposal on cognitive penetrability of perception suggests that the purpose of attention is to predict, to transform incoming sensory energy “into a useful form for a particular perceptual goal” for minimizing “global prediction error” (Lupyan, 2015, pp. 553, 564). On the debate of whether perception is cognitively penetrable via attention, a significant divide lies in the domain of evidence supporting each view. The passive filter/gate-keeper view has been built on research findings on the spatial processing of attention (e.g., the spot light metaphor of attention; Posner et al., 1980; Eriksen and Yeh, 1985; Awh and Pashler, 2000). In contrast, the more recent cognitive-penetrable view of perception is supported by much evidence on the non-spatial processing of attention (e.g., Egly et al., 1994; Moore et al., 1998; Lupyan, 2008; Lupyan and Spivey, 2010; Baldauf and Desimone, 2014). It is possible that the space-based attention and object-based attention are two streams of attentional processing that serve distinct purposes and are based on different mechanisms. Thus, the non-spatial processing of attention could be much more open than spatial attention to cognitively penetration of perception.

However, is spatial attention completely immune to this cognitive penetration? One approach to this question is to examine whether the attentional modulation in pre-cueing takes place before perceptual processing (e.g., Pylyshyn, 1999; Lupyan, 2015). Another way to answer the question is by making a distinction between early and late vision (e.g., Raftopoulos, 2009). Here we propose a third way to look at the question, that is to explore potential interaction between space-based and non-space based attentional mechanisms. For example, it is possible that the size of the area that attentional selection is operating on and the object representation in attention are related. When we are looking for an object in a larger area, given extended space for simultaneous visual processing, we could be working with a more simplified representation of the object with reduced dimensions of features to allow efficient process. This would especially make sense when we consider attentional processing across the visual field as the visual periphery would only allow coarse processing. For instance, when one looks for a friend on the street without much idea of where the friend might be, the attentional mechanism may be just based on clothing color and general body shape. If the individual knows which street corner the friend is at, he/she could use a more detailed representation for target detection. A smaller area may lead to more concentrated spatial attention, however, with a more detailed object representation, target detection may not be faster. This could be a possible reason why we observed no change on target detection at an eccentricity of 10° across pre-cue conditions in our Experiment 1 (particularly Experiment 1A when significant changes were observed at eccentricities of 20° and 30°). Nevertheless, it is speculative at this stage and our current experiments were not specifically designed to evaluate this hypothesis. Future experimental work, exploring attentional processing across an extended visual area, is needed to carefully examine this speculation.

### Eccentricity as an Important Factor in Understanding Attention

In our experiments, the size of the stimuli was kept constant across eccentricities. Therefore, we cannot distinguish between impacts from cortical and subcortical mechanisms on our eccentricity effect. One way to separate these impacts is to increase stimulus size as its location becomes more peripheral (i.e., M-scale the stimuli; Rovamo and Virsu, 1979). Several studies have attempted to contrast the results with and without M-scaling (Carrasco et al., 2003; Bao et al., 2013; Staugaard et al., 2016), and found that eccentricity effects were not completely eliminated by M-scaling, suggesting that the eccentricity effects were a combination of cortical magnification and other attentional mechanisms. It is possible that the eccentricity effects found in our experiments were also a combination of various neurophysiological and attentional mechanisms. Further examination is needed to isolate the impact from each individual mechanism.

In many tasks, attention must operate over a very large visual area to achieve superior performance in many daily tasks. For example, older adults are generally less able to identify important events in a cluttered visual environment across the visual field and this decline in selective attention can lead to poorer driving performance (Ball et al., 1990, 1993; Bedard et al., 2006) and higher risks of falls (Lajoie et al., 1996; Broman et al., 2004; Owsley and McGwin, 2004). During spatial navigation, blocking a participant’s peripheral vision leads to severe impairment in wayfinding (Fortenbaugh et al., 2006). However, in the empirical efforts to understand pre-cueing effects in the laboratory, visual attentional processing, especially endogenous attention (i.e., the cognitive driven pre-cueing), had rarely be examined over an eccentricity of 20°. Given the intense coupling between attention and saccades (Sheliga et al., 1994; Hoffman and Subramaniam, 1995; Peterson et al., 2004), and a significant change in the saccadic characteristics at 20° of eccentricity (e.g., plateau of amplitude; Bahill et al., 1975), exploring visual attention across a large visual area that expands beyond 40° of visual angle is important. Our study found that effect of pre-cueing (using endogenous spatial cues) was in general greater at a larger eccentricity. This highlight the capability of our attentional system so we can intensely utilize our visual periphery for many daily tasks despite its sensory limitations. An important aspect that differs our second experiment with many earlier attentional studies on of endogenous direction cues (e.g., Bashinski and Bacharach, 1980; Posner, 1980) is the examination of eccentricity effect across an extended visual area (60° of visual angle). Our results showed that attentional shift is possible to a very distant area (at least up to 30° of amplitude) within a relatively brief period of time (even an 80 ms interval between the onsets of a cue and stimulus significantly speeded response). Although a shift of gaze larger than 20° is often accompanied by a head movement, our capability to quickly orient attention to relatively peripheral areas may provide the benefit to improve our overall evaluation of the visual stimuli and more accurately determine where the gaze will move to.

## Ethics Statement

This study was carried out in accordance with the recommendations of Guide for Informed Consent and Guidelines for Ethical Conduct in Participant Observation, Research Ethics Boards of the University of Toronto with written informed consent from all subjects. All subjects gave written informed consent in accordance with the Declaration of Helsinki. The protocol was approved by the Social Sciences, Humanities and Education Research Ethics Board of the University of Toronto.

## Author Contributions

JF and IS contributed to the concept of the study. JF conducted the experiment and analyzed the data, under the supervision of IS. JF and IS contributed to the interpretation of the results and preparing the manuscript.

## Conflict of Interest Statement

The authors declare that the research was conducted in the absence of any commercial or financial relationships that could be construed as a potential conflict of interest.
